# Proteomic analysis of elite soybean Jidou17 and its parents using iTRAQ-based quantitative approaches

**DOI:** 10.1186/1477-5956-11-12

**Published:** 2013-03-26

**Authors:** Jun Qin, Feng Gu, Duan Liu, Changcheng Yin, Shuangjin Zhao, Hao Chen, Jianan Zhang, Chunyan Yang, Xu Zhan, Mengchen Zhang

**Affiliations:** 1 National Soybean Improvement Center Shijiazhuang Sub-Center. North China Key Laboratory of Soybean Biology and Genetic Improvement, Ministry of Agriculture. Cereal & Oil Crop Institute, Hebei Academy of Agricultural and Forestry Sciences, Shijiazhuang 050031, P.R. China; 2 Geochemical Environmental Research Group, Texas A&M University, 833 Graham Road, College Station, TX 77845, USA; 3 Beijing Protein Innovation, B-8, Beijing Airport Industrial Zone, Beijing 101318, P.R. China; 4 Institute of Millet Crops, Hebei Academy of Agriculture and Forestry Sciences, Shijiazhuang 050031, P.R. China

**Keywords:** Soybean, iTRAQ, Proteomic

## Abstract

**Background:**

Derived from Hobbit as the female parent and Zao5241 as the male parent, the elite soybean cultivar Jidou17 is significantly higher yielding and shows enhanced qualities and stronger resistance to non-biological stress than its parents. The purpose of this study is to understand the difference in protein expression patterns between Jidou17 and its parental strains and to evaluate the parental contributions to its elite traits.

**Results:**

Leaves (14 days old) from Jidou17 and its parental cultivars were analysed for differential expressed proteins using an iTRAQ-based (isobaric tags for relative and absolute quantitation) method. A total of 1269 proteins was detected, with 141 and 181 proteins in Jidou17 differing from its female and male parent, respectively. Functional classification and an enrichment analysis based on biological functions, biological processes, and cellular components revealed that all the differential proteins fell into many functional categories but that the number of proteins varied greatly for the different categories, with enrichment in specific categories. A pathway analysis indicated that the differentiated proteins were mainly classified into the ribosome assembly pathway. Protein expression clustering results showed that the expression profiles between Jidou17 and its female parent Hobbit were more similar than those between Jidou17 and its male parent Zao5241 and between the two parental strains. Therefore, the female parent Hobbit contributed more to the Jidou17 genotype.

**Conclusions:**

This study applied a proven technique to study proteomics in 14-day-old soybean leaves and explored the depth and breadth of soybean protein research. The results provide new data for further understanding the mechanisms of elite cultivar development.

## Introduction

Traditional plant breeders developing new cultivars mainly rely on phenotypes, which, for soybean breeding, often include such agricultural characteristics as growth, biomass, yield, resistance to disease, and tolerance to environmental stress. With the introduction of physiological markers, photosynthesis, matter accumulation, and stress-related enzymes are now being evaluated in breeding programmes. The targeted phenotypes and physiological characters generally correspond to functional proteins participating in photosynthesis, protein synthesis, maintenance of the intracellular environment, and functional stability. These proteins play critical roles in important pathways and are clearly related to the resulting crop yield and quality. Therefore, it is useful to identify key proteins involved in these pathways and to understand their contributions and relationships through expression pattern analysis.

Crop yields ultimately depend on the photosynthesis ability of plants [1], essentially corresponding to the size and efficiency of the photosynthetic system [2]. Because all of the metabolic processes involved in photosynthesis primarily occur in the leaves, these organs represent one of the primary organs subjected to biological study. Soybean is an important economic crop, and the selection of filial generations during breeding is conducted based on the evaluation of important agronomic traits. Therefore, a newly bred cultivar always possesses some elite traits from it parents. Exploration of the development of different phenotypes and differential protein expression patterns could reveal some of the mechanisms involved in cultivar development.

The elite soybean cultivar Jidou17 was derived from a Hobbit×Zao5241 cross and was developed via sexual hybridisation through multiple years of directional selection for high oil production and plant type. In the Huang-Huai-Hai regional combined cultivar competition, this cultivar was awarded first place in three consecutive years, from 2008–2010 and was also awarded first place in the 2010–2011 northern China spring sowing area-wide experiment and Huang-Huai-Hai summer sowing region-wide experiment. Therefore, the insights obtained through proteomic analyses of Jidou17 and its parents will have important implications.

A number of researchers have studied the protein and physiological characteristics of soybean leaves. The comparison of the leaves of three groups of soybean plants (semiwild, semicultivated, and cultivated) revealed that the amount of soluble carbohydrates and proteins increased with the process of domestication [3], with the semiwild group being less domesticated than the semicultivated group. The same set of soybeans was tested to determine the activities of peroxidase (POD) and superoxide dismutase (SOD), and both activities were observed to be higher in the leaves of plants with higher domestication levels. Wang studied the physiological characters of soybean leaves exposed to soybean aphid (*Aphis glycines* Matsumura) stress [4], measuring the contents of soluble and insoluble proteins, soluble carbohydrates, and the activities of two defence enzymes, peroxidase (POD) and catalase (CAT). Wen et al. (1999) examined the diurnal variations in the photosynthetic efficiency, nitrate reductase activity, and soluble protein levels in the leaves of three soybean cultivars, indices that were found to be correlated with the diurnal cycle and photosynthetic rate [5]. Wang and colleagues used soybean primary leaves to study the variations in the autophosphorylation and enzyme activity of a plasma membrane kinase during the leaf aging process [6]. However, a systematic analysis of soybean leaf protein expression in specific developmental stages has not yet been reported.

As the experimental approaches employed in this field have expanded from conventional analyses of single markers (*e.g.,* SOD and POD isoenzymes) to multiple markers, more aspects have been studied simultaneously, and the research has been extended to proteins with unknown functions. To examine differentially expressed proteins, researchers previously had to rely on 2D gels to detect differentially expressed spots or DIGE with fluorescent markers [7-9]. In efforts to increase sensitivity and accuracy, mass spectrometry has also been employed for protein identification. A combination of gel-based and gel-free proteomic techniques has also been used for the identification of soybean plasma membrane proteins under flooding [10,11] or osmotic stress [12], suggesting that these two methods are complementary to one another for protein identification. However, the methods employed for protein identification are not usually organism specific and can be applied to a wide range of organisms.

iTRAQ (isobaric tags for relative and absolute quantitation) is a non-gel-based protein identification and quantification technique involving isotope labelling that was recently developed for the absolute and relative quantification of proteins. iTRAQ has become one of the major quantification tools used in differential proteomic research [13]. For years, 2D electrophoresis was one of the main methods applied to the study of differences in protein content in biological samples [7]. In this approach, samples are labelled with different dyes and subjected to differential in-gel electrophoresis (DIGE); the differentially expressed gel spots are then excised and analysed using mass spectrometry. In contrast to this older method, the iTRAQ technique involves a combination of multi-dimensional liquid chromatography and tandem mass spectrometry. iTRAQ can be used to analyse several samples within one assay, and no gel electrophoresis is necessary, which avoids the loss of either small molecules (<10 kD) or large molecules (>200 kD). Furthermore, because there is no electrophoresis involved in iTRAQ, this method can reduce the analytical bias caused by different hydrophilicities, abundances, or isoelectric points, and the applied isotope labelling increases the sensitivity and accuracy. Many studies have demonstrated that iTRAQ is an effective method for examining proteins that show differential expression under different physiological conditions or pathological conditions. Ross [14] pioneered the application of iTRAQ to wild-type and two mutant *Saccharomyces cerevisiae* samples and revealed differential protein expression patterns. The iTRAQ technique is currently mainly employed in medical research, and there are few reports of its use in plant applications. For example, a proteomic analysis of *Thermobifida fusca* membrane proteins under conditions involving a lack of cellulose was reported based on the iTRAQ method [15], and Ping Lan [16] revealed the protein profile of *Arabidopsis* roots under iron homeostasis using iTRAQ.

In the present study, iTRAQ was applied for the proteomic analysis of the 14^th^ leaf developmental stage of soybean to expand the depth and breadth of our knowledge of soybean protein expression. The aims of the study were as follows: 1) to identify differentially expressed proteins between Jidou17 and its parental strains; 2) to compare the obtained protein expression patterns; and 3) to reveal the developmental basis of the of the elite traits of Jidou17.

## Results

### Proteomic analysis

**Figure 1:**
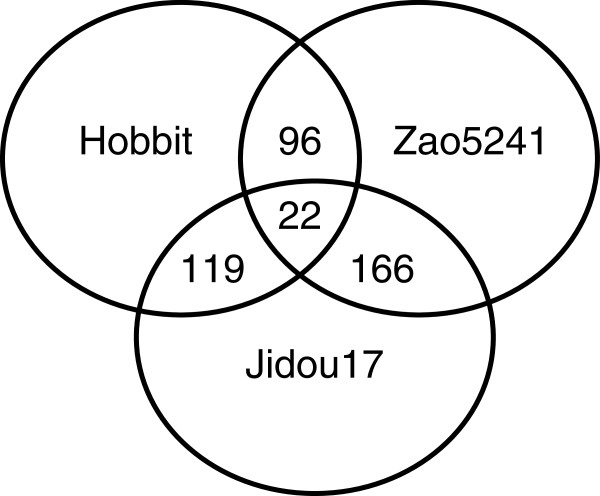
Venn diagram showing the differentially expressed proteins identified in Jidou17 and its parents.

The iTRAQ method was applied for the proteomic analysis of 14-day-old leaves from Jidou17, its female parent (Hobbit), and its male parent (Zao5241). A total of 1269 non-redundant proteins were detected; the differentially expressed proteins are shown in a Venn diagram in Figure 1. When the expression level of a protein showed a difference corresponding to a >2-fold or <0.5-fold change in abundance compared to the control strain, that protein was considered to be differentially expressed. When Jidou17 was considered the control strain, the female parent displayed 141 differentially expressed proteins of which 75 presented higher expression, whereas 84 showed lower expression. In contrast, the male parent exhibited 188 differentially expressed proteins of which 61 displayed higher expression, with 127 showing lower expression. Thus, the male parent (Zao5241) presented more differentially expressed proteins than the female parent (Hobbit). When the male parent Zao5241, was used as the control, the female parent exhibited 118 differentially expressed proteins of which 24 showed higher expression and 94 lower expression. In addition, there were 22 proteins that were differentially expressed among all three cultivars.

### Protein expression pattern analysis between Jidou17 and its parental cultivars

**Figure 2:**
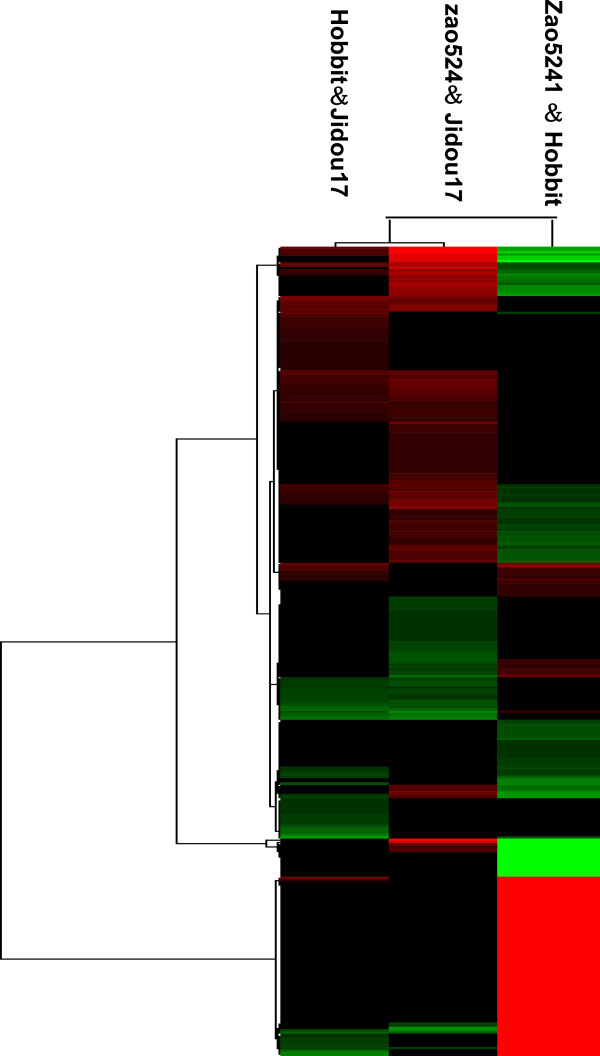
**Cluster map comparing the protein expression patterns of Jidou17, Zao5241, and Hobbit.** Red indicates higher expression, green indicates lower expression, and black indicates the same expression levels in the two strains.

The examination of differentially expressed proteins between a filial generation and its parents aids in an understanding of the relevance of protein expression profiles and agronomic traits. A cluster analysis of the proteins identified in the Jidou17, Zao5241, and Hobbit cultivars was conducted using Cluster 3.0 software (Michael Eisen, Stanford University), and the proteins were grouped depending on their expression level. The protein expression patterns of Hobbit and Jidou17 shared a higher similarity than those of Zhao5241 and Jidou17 (Figure 2), and the similarity of Jidou17 with both of its parents was higher than the similarity between Zao5241 and Hobbit. Therefore, the protein expression pattern of Jidou17 was closer to that of the female parent.

The proteins that were differentially expressed between Jidou17 and its parental strains were classified into seven categories (Table 1). Category 1 corresponded to differentially expressed proteins specific to Jidou17 and Hobbit. Within this category, the expression levels observed in Zao5241 were between those of Jidou17 and Hobbit. Category 1 had two subcategories in which the expression levels in Hobbit were higher or lower than those in Jidou17. Category 2 comprised the proteins that were differentially expressed only between Jidou17 and Zao5241. Category 2 was similar to Category 1. Within this category, the expression levels in Hobbit were between those found in Jidou17 and Zao5241. Category 2 also included two subcategories, comprising proteins of a lower or higher expression level in Zao5241 than Jidou17. Category 3 was composed of the proteins that were differentially expressed between the two parental strains that were not differentially expressed between one of the parents and Jidou17. The expression levels of the Category 3 proteins in Jidou17 were between those in the two parental strains. Category 4 proteins were differentially expressed between the two parental strains and differentially expressed between Jidou17 and Hobbit; these proteins exhibited similar expression levels in Jidou17 and Zao5241. Accordingly, Category 5 proteins were differentially expressed between the two parental strains and differentially expressed between Jidou17 and Zao5241; these proteins displayed similar expression levels in Jidou17 and Hobbit. Category 6 consisted of proteins that were differentially expressed between Jidou17 and both parental strains but were not differentially expressed between the two parental strains. Lastly, Category 7 proteins were differentially expressed between all three strains.

**Table 1:** The protein expression pattern.

**The protein expression pattern**	**The categories of differentially expressed proteins**	**The numbers of differentially expressed proteins**
I	The differentially expressed proteins specific to Jidou17 and Hobbit	52
II	the proteins that differentially expressed only between Jidou17 and Zao5241	64
III	proteins that differentially expressed in Hobbit and Zao5241	29
IV	proteins were differentially expressed between two parental strains and also differentially expressed between Jidou17 and Hobbit	17
V	proteins were differentially expressed between two parental strains and differentially expressed between Jidou17 and Zao5241	52
VI	proteins differentially expressed between Jidou17 and both parental strains.	52
VII	proteins were differentially expressed in all three strains.	20

### Classification into functional groups

**Figure 3:**
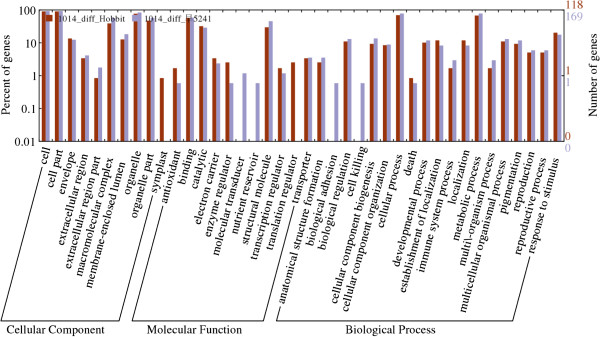
Functional categorisation of the proteins that were differentially expressed between Jidou17 and its parental strains.

To reveal the functions of the proteins that were differentially expressed in Jidou17, these proteins were classified into 3 large groups and 40 subgroups depending on their functional annotation (Figure 3). The 3 large groups corresponded to molecular functions, biological processes, and cellular components. On the left side of the Y axis in Figure 3, the most populated subgroup was considered to represent 100%, and the percentages of other subgroups were determined relative to that. The proteins that were differentially expressed between Jidou17 and Hobbit were distributed among 36 subgroups, whereas those that were differentially expressed between Jidou17 and Zao5241 were distributed into 38 subgroups. Both of these categories displayed proteins that were distributed among almost all of the subgroups. Two subgroups, the symplast and translation regulator subgroups, were found among the proteins that were differentially expressed between Jidou17 and Hobbit but not for those showing differential expression between Jidou17 and Zao5241. In contrast, 4 subgroups, molecular transducers, nutrient reservoirs, biological adhesion, and cell killing, were found for the proteins that were differentially expressed between Jidou17 and Zao5241 but not for those showing differential expression between Jidou17 and Hobbit.

### Protein enrichment analysis

**Figure 4:**
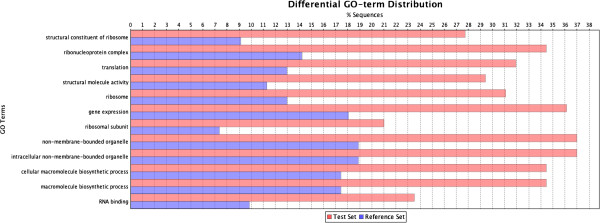
**GO term distribution of the enriched proteins that were differentially expressed between Jidou17 and Hobbit.** The blue stripes indicate the expected protein number for each functional group; the red stripes represent the actual detected protein number.

**Figure 5:**
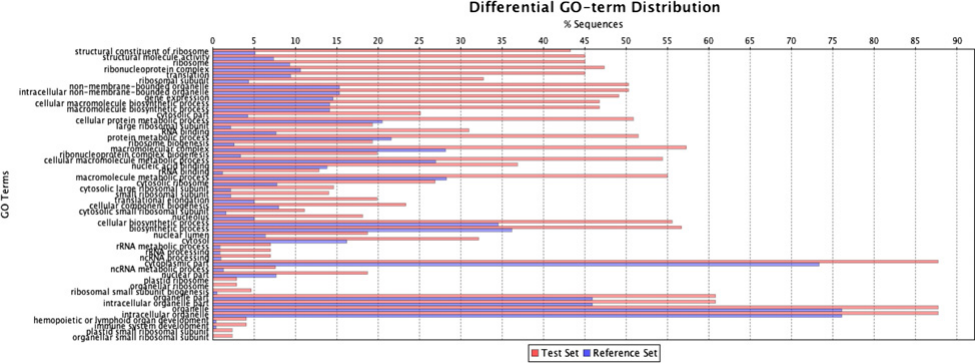
**GO term distribution of the enriched proteins that were differentially expressed between Jidou17 and Zao5241.** The blue stripes indicate the expected protein number for each functional group; the red stripes represent the actual detected protein number.

To determine whether the differentially expressed proteins were enriched in certain functional groups, an enrichment analysis was conducted using Blast2GO. The 1269 identified proteins were classified into a total of 1674 groups, and this classification was also applied to the differentially expressed proteins. The distribution of all proteins and the distribution of differentially expressed proteins were compared to determine whether the differentially expressed proteins were enriched for certain categories (Figures 4 and 5). Three different significance parameters were applied to control for false positives. Figures 4 and 5 display the significant GO terms, ranked according to their significance level; the blue stripes represent the expected protein number for each functional group, and the red stripes indicate the actual detected protein number. The proteins that were differentially expressed between Jidou17 and Hobbit and those that were differentially expressed between Jidou17 and Zao5241 displayed rather different enrichment distributions. The proteins that were differentially expressed between Jidou17 and Hobbit were enriched in 12 functional categories (Figure 4) of which cellular components represented 5 categories, molecular functions represented 3 categories, and biological processes represented 4 categories. In contrast, the proteins that were differentially expressed between Jidou17 and Zao5142 were enriched in 51 functional categories (Figure 5) of which cellular components accounted for 25 categories (most of which were related to ribosomes), molecular functions accounted for 5 categories (most of which were related to ribosomal structure and function), and biological processes accounted for 21 categories. Some proteins related to biological process were also related to protein metabolism.

### Pathway analysis

**Figure 6:**
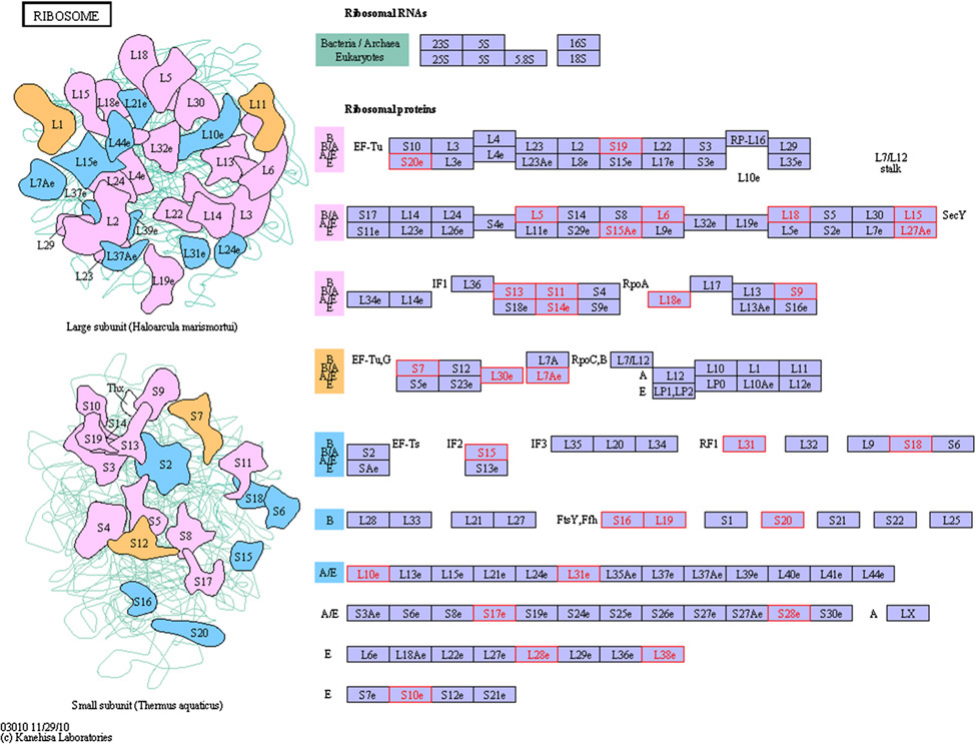
**Differentially expressed proteins between Jidou17 and Hobbit enriched in the ribosomal assembly pathway.** Red indicates differentially expressed proteins.

**Figure 7:**
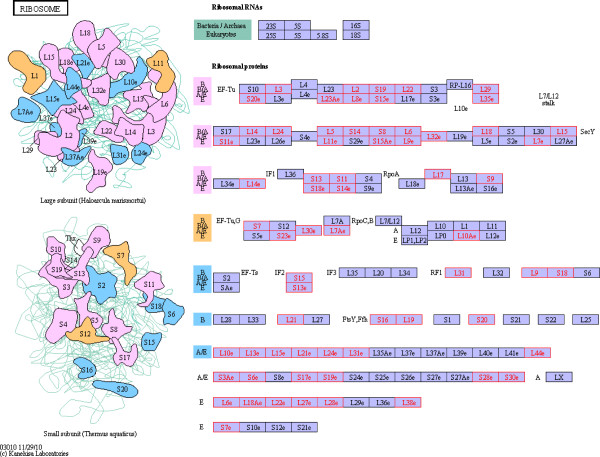
**Differentially expressed proteins between Jidou17 and Zao5241 enriched in the ribosomal assembly pathway.** Red indicates differentially expressed proteins.

We compared all of the 1269 identified proteins with *Arabidopsis thaliana* proteins, and 1238 matches were found. *Arabidopsis thaliana* is a model species for plant molecular biology research; its genome has been sequenced, and intensive research has been conducted on proteomic and molecular pathways in *Arabidopsis*. Of the proteins that were differentially expressed between Jidou17 and Hobbit, 24 enriched in the ribosomal assembly pathways described for *Arabidopsis* (Figure 6), and 53 of the proteins that were differentially expressed between Jidou17 and Zao5241 enriched in this category (Figure 7). Because ribosomes are the sites of protein synthesis, the increased expression of these proteins may have improved protein synthesis in Jidou17.

### Verification of the differential expression of some important proteins using antibodies

**Figure 8:**
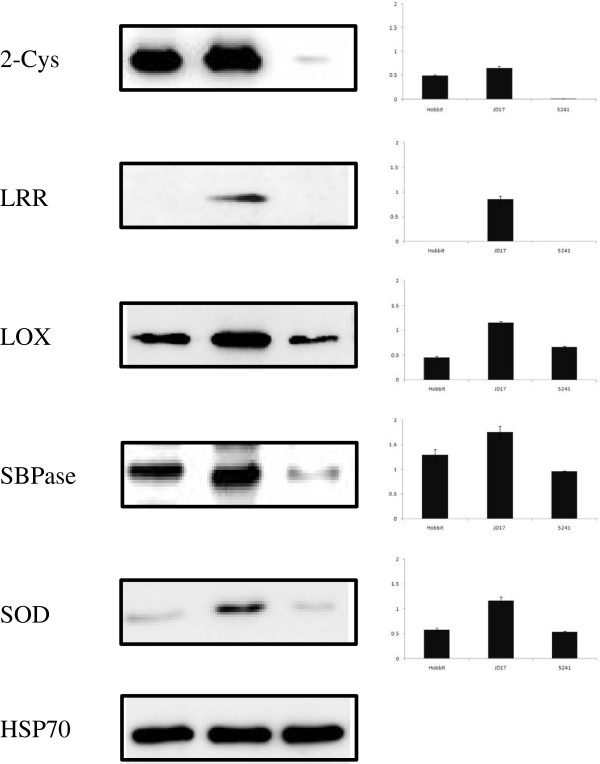
Western blotting detection of soybean proteins 2-Cys, LRR, LOX, SBPase, SOD.

Western blotting was performed to verify the expression of targeted proteins identified by the iTRAQ analysis at the 14^th^ leaf developmental stage. We selected five differentially expressed proteins 2-Cys, LRR, LOX, SBPase and SOD. and subjected the same protein samples that were used for iTRAQ analysis to western blotting. The stability of the expression of these proteins was analysed using geNorm and Microcal Origin 6.0 software. The western blots for 2-Cys, LRR, LOX, SBPase and SOD showed results that were consistent with the iTRAQ data (Figure 8). Jidou17 exhibited more highly expressed proteins than its parents. These results demonstrated the satisfactory quality of our experimental procedures and data.

## Discussion

### Seedling protein expression model and parental similarities

This study analysed the differentially expressed protein profiles between Jidou17 and its parental strains. The number of proteins that were differentially expressed between Jidou17 and its female parent (Hobbit) was 141, which was lower than for its male parent (Zao5241). Classification of the proteins showed that the proteins that were differentially expressed between Jidou17 and Hobbit were distributed into 36 subgroups; those that were differentially expressed between Jidou17 and Zao5241 were distributed among 38 subgroups. Thus, the proteins that were differentially expressed between Jidou17 and Hobbit were distributed in fewer subgroups when compared to Zao5241. When the obtained protein expression patterns were compared**,** those of Jidou17 and Hobbit showed higher similarity than those of Jidou17 and Zao5241, and the similarity was higher between Jidou17 and both parental strains than between the two parental strains themselves. The protein expression pattern detected in Jidou17 at this growth stage was close to that of Hobbit. Regarding the proteins associated with the ribosomal assembly pathway, 24 of the proteins that were differentially expressed between Jidou17 and Hobbit fell into this category, as did 53 that were differentially expressed between Jidou17 and Zao5241. The above results are in agreement with each other, providing further support that our overall results are reliable.

### Notable proteins that were differentially expressed

There were some noteworthy proteins among the proteins that were differentially expressed between Jidou17 and its parents, including ferredoxin/thioredoxin reductase, acid phosphatase, oxidoreductase, peroxidase, superoxide dismutase (SOD), At2g46140-like desiccation-related protein, and translation extension factors. This group also included enzymes related to fatty acid synthesis and lipoxygenase (LOX). Regarding the quality of soybean products, lipoxygenase is an anti-nutritional factor that affects the soybean food, storage, and processing qualities. Lipoxygenase also affects development, maturation, and aging of soybean plants [17] and stimulates seedling growth, increases stress resistance, and boosts defence reactions [18,19]. LOX also serves as a storage protein [17] and participates in lipid and protein relocation [20,21]. D4N5G3, a RuBisCO activase, was also identified as being differentially expressed; RuBisCO is one of the most important enzymes in leaves because it plays an important role in the Calvin cycle [22]. Another differentially expressed protein, O49856, is a ferredoxin-thioredoxin reductase that catalyses the light-dependent activation of several photosynthetic enzymes [23,24]. Though not listed here, there were additional differentially expressed proteins associated with other critical pathways. As a future research direction, we hope that these results will help direct breeding practices.

## Materials and methods

### Materials

Jidou17, Hobbit, and Zao5241 were grown in a greenhouse at Shijiazhuang (114°26’E, 38°03’N). When a fully developed trifoliolate leaf node displayed unrolled leaflets (V2), the first fully developed trifoliolate leafs were collected from seeding exhibiting equivalent growth on the 14^th^ day. The leaves were frozen in liquid nitrogen and stored at −80°C.

### Protein extraction

Total proteins were extracted using the cold acetone method. Liquid nitrogen and polyvinylpolypyrrolidone (at approximately 10% of the sample volume) were added, and the samples were then ground to disrupt the cells. Next, 10% trichloroacetic acid (TCA) in acetone was added to the samples, followed by incubation at −20°C for 2 h and centrifugation at 30,000 × g at 4°C for 30 minutes. The supernatant was discarded without disturbing the white pellet. To reduce the acidity, the pellets were resuspended with cold acetone and centrifuged again at 20,000 × g for 30 minutes at 4°C; this acetone washing step was repeated 3 times. Each pellet was resuspended in 1 ml of protein extraction reagent [8 M urea, 4% (w/v) CHAPS, 30 mM HEPES, 1 mM PMSF, 2 mM EDTA, and 10 mM DTT] with sonicated. The samples were then centrifuged at 20,000 × g for 30 min at 4°C, and the pellets were discarded, as the proteins were in the supernatant at this step. The protein concentration was determined with the 2-D Quant Kit (General Electric Company, USA), and the samples were stored at −80°C. SDS-PAGE was performed to verify the protein quality and concentration.

### Protein digestion

A 100-μl aliquot of each protein sample was combined with an equal volume of tetraethylammonium bicarbonate (TEAB), pH 8.5, followed by treatment with trypsin (3.3 μg trypsin/100 μg total protein). The proteins in TEAB were digested with trypsin at 37°C for 24 h, and the solvent was removed using a SpeedVac. MALDI TOF/TOF analysis was conducted to test the efficiency of digestion.

### iTRAQ labelling

The iTRAQ labelling procedure was performed following the manual provided in the iTRAQ labelling kit (Applied Biosystems), unless otherwise specified. For each protein sample, 100 μg of protein was denatured, and the cysteines were blocked as described in the iTRAQ protocol. The protein samples were then digested with 5 μg of sequencing-grade modified trypsin (Promega, Madison, WI) at 37°C for 36 h. The digested samples were dried in a centrifugal vacuum concentrator, and the protein pellets were dissolved in 30 μl of 50% TEAB (Sigma, St. Louis, MO) together with 70 μL of isopropanol and labelled with the iTRAQ reagents according to the protocol of 8-plex iTRAQ labelling kit. The trypsin-digested samples were analysed via MALDI-TOF-TOF to ensure complete digestion. iTRAQ tags 113–121 were added to the digested protein samples during labelling. The iTRAQ-labelled samples were then pooled and subjected to strong cation exchange (SCX) fractionation.

### Strong cation exchange fractionation

The labelled samples were fractionated using a high-performance liquid chromatography (HPLC) system (Shimadzu, Japan) connected to an SCX column (Luna 5-μm column, 4.6 mm I.D. × 250 mm, 5 μm, 100 Å; Phenomenex, Torrance, CA). The retained peptides were eluted using Buffer A (10 mM KH_2_PO_4_ in 25% ACN, pH 3.0) and Buffer B (2 M KCl, 10 mM KH_2_PO_4_ in 25% ACN, pH 3.0), and the fractions were collected in 1.5-ml microfuge tubes. The flow rate was set at 1 mL/min. The following gradient was applied: 30 min of 100% buffer A; from 30~31 min, the buffer B concentration was increased to 5%; from 31~46 min, the buffer B concentration was increased to 30%; from 46~51 min, the buffer B concentration was increased to 50% and then maintained for 5 min; and from 55–61 min, the buffer B concentration was increased to 100%. All solutions were freshly prepared and filtered through a 0.22-μm membrane. Fractions were collected every minute starting at 31 min after sample injection, and a total of 38 fractions were collected. For those fractions with a high salt content, the salt was removed using a Strata-X 33-μm Polymeric Reversed Phase column. The eluted fractions were dried in a vacuum concentrator and then dissolved with 0.1% formic acid prior to reverse-phase nLC-tandem mass spectrometry.

### Reverse-phase nanoliquid chromatography/tandem MS (LC-MS/MS)

The peptide content in each fraction was equalised prior to injection into the Nano-LC system. For the MALDI-TOF/TOF analysis, the SCX peptide fractions were pooled together to obtain 17 fractions to reduce the peptide complexity. A 10-μl aliquot of each fraction was injected twice into the Proxeon Easy Nano-LC system. The peptides were separated using a C18 analytical reverse-phase column at a solvent flow rate of 300 nL/min (Solution A, 5% acetonitrile/0.1% formic acid; Solution B, 95% acetonitrile/0.1% formic acid) over 120 min. A linear LC gradient profile was used to elute the peptides from the column. After sample injection, the column was equilibrated with 5% Solution B for 10 min, and the following gradient schedule was then initiated: 45% Solution B at 80 min; 80% Solution B at 85 min and maintained for 15 min; and 5% Solution B at 105 min and held for 15 min before ramping back down to the initial solvent conditions. The fractions were analysed using a hybrid quadrupole/time-of-flight MS (MicroTOF-Q II, Bruker, Germany) with a nano-electrospray ion source. The data were collected and analysed using Data Analysis Software (Bruker Daltonics, Bremen, Germany). The MS/MS scans were recorded from 50–2000 m/z. Nitrogen was used as the collision gas. The ionisation tip voltage and interface temperature were set at 1250 V and 150°C, respectively.

### Desalting via C18 reverse-phase chromatography

A C18 solid-phase extraction cartridge was activated using 1 ml methanol at a speed of 2–3 drops per second. The column was then balanced with 5% ACN at a speed of 1 drop per second. The column was then washed with MilliQ H_2_O, and the outflow sample was collected to prevent the loss of non-column-binding peptides. The obtained peptides were desalted with 1 ml 5% ACN at a speed of 1 drop per second. The elution was performed twice using 500 μl of ACN at a speed of 1 drop per second. The ACN was removed using a SpeedVac, and the peptides were resuspended in 0.1% formic acid.

### Characterisation of digested peptides via micro-TOF-Q (Bruker)

Similar peptides were collected, freeze-dried, and resuspended in 20 μl 0.1% formic acid. Micro-TOF-Q was used to evaluate the quantity of the peptides.

### Data analysis

All of the mass spectrometry data were collected using Bruker Daltonics micrOTOFcontrol and processed and analysed using the Data Analysis Software. The Uniprot database was downloaded and integrated into the Mascot search engine, version 2.3.01, through its database maintenance unit. The parameters were set as follows: trypsin was specified as the digestion enzyme, cysteine carbamidomethylation as a fixed modification, iTRAQ8Plex on the N-terminal residue, iTRAQ 8Plex on tyrosine (Y), iTRAQ 8Plex on lysine (K), glutamine as pyroglutamic acid, and oxidation on methionine (M) as a variable modification. The tolerance settings for peptide identification in the Mascot searches were 0.05 Da for MS and 0.05 Da for MS/MS. The Mascot search results were exported into a DAT FILE and normalised and quantified using Scaffold version 3.0 Software.

### Functional analysis of differentially expressed proteins

To predict the functions of the differentially expressed proteins, we analysed the proteins with regard to three aspects. First, we categorised the proteins functionally using the WEGO (web Gene Ontology Annotation Plot) web service (http://wego.genomics.org.cn/cgi-bin/wego/index.pl). The consensus or exemplar sequences of the proteins were then subjected to BLAST searches against the corresponding soybean sequence database, and the top hits were selected using an E-value cut-off of 10–30. Next, we performed a functional category gene enrichment test using Blast2GO to determine whether the differentially expressed proteins were significantly enriched in any functional subcategories. The 1269 proteins were used as the default background distribution of each functional subcategory. Three different significance parameters were used to control for false positives: the false discovery rate (FDR), family-wise error rate (FWER), and single-test P-value (Fisher P-value). An FDR significance threshold of 0.05 was selected. Lastly, we allocated the differentially expressed proteins to biological pathways using the KEGG (Kyoto Encyclopedia of Genes and Genomes) resource (http://www.genome.jp/kegg/)[25-27].

### Western blot analysis

The same samples used in the iTRAQ analysis were also used for a western blot analysis. Five differentially expression proteins were selected, and equal amounts of the protein samples from Jidou17 and its parental cultivars were separated via SDS–PAGE and electrotransferred to a PVDF membrane (Millipore Corporation, Bedford, MA, USA) at 100 V for 60 min. The membrane was then immersed in 5% non-fat milk in TTBS solution [0.2 M Tris–HCl (pH 7.6), 1.37 M NaCl, and 0.1% Tween-20] for 1 h at room temperature. The proteins were incubated with the corresponding polyclonal antibodies in 5% non-fat milk in TTBS solution for 3 h at room temperature and then subjected to three 5-min rinses in TTBS solution. The membrane was next incubated with a horseradish peroxidase-conjugated goat anti-rabbit antibody (Beijing Protein Innovation, Beijing, China) for 1 h at room temperature and subjected to three 5-min rinses in TTBS solution. The blot was then developed with the SuperECL Plus kit (Applygen, Beijing, China), and the signal was detected with X-ray film.

## Competing interests

The authors declare that they have no competing interests.

## Authors’ contributions

JQ contributed to the project idea, performed the experiments and data analysis, and composed the draft of the manuscript. FG performed data acquisition and interpretation. DL, CY, SZ, and HC contributed to the proteomic analysis. CY and XZ participated in the supervision of the study. MZ obtained grant funding. All authors have read and approved the final manuscript.
